# Systemic cross-talk between brain, gut, and peripheral tissues in glucose homeostasis: effects of exercise training (CROSSYS). Exercise training intervention in monozygotic twins discordant for body weight

**DOI:** 10.1186/s13102-021-00241-z

**Published:** 2021-02-24

**Authors:** Marja A. Heiskanen, Sanna M. Honkala, Jaakko Hentilä, Ronja Ojala, Riikka Lautamäki, Kalle Koskensalo, Martin S. Lietzén, Virva Saunavaara, Jani Saunavaara, Mika Helmiö, Eliisa Löyttyniemi, Lauri Nummenmaa, Maria C. Collado, Tarja Malm, Leo Lahti, Kirsi H. Pietiläinen, Jaakko Kaprio, Juha O. Rinne, Jarna C. Hannukainen

**Affiliations:** 1 Turku PET Centre, University of Turku, P.O. Box 52, FIN-20521 Turku, Finland; 2 Heart Centre, Turku University Hospital, Turku, Finland; 3 Department of Medical Physics, Turku University Hospital, Turku, Finland; 4 Division of Digestive Surgery and Urology, Turku University Hospital, Turku, Finland; 5 Department of Biostatistics, University of Turku, Turku, Finland; 6 Department of Psychology, University of Turku, Turku, Finland; 7 Institute of Agrochemistry and Food Technology-National Research Council (IATA-CSIC), Valencia, Spain; 8 Functional Food Forum, University of Turku, Turku, Finland; 9 A.I. Virtanen Institute for Molecular Sciences, University of Eastern Finland, Kuopio, Finland; 10 Department of Future Technologies, University of Turku, Turku, Finland; 11 Obesity Research Unit, Research Program for Clinical and Molecular Metabolism, Faculty of Medicine, University of Helsinki, Helsinki, Finland; 12 Abdominal Center, Obesity Center, Endocrinology, University of Helsinki and Helsinki University Central Hospital, Helsinki, Finland; 13 Institute for Molecular Medicine Finland FIMM, HiLIFE, University of Helsinki, Helsinki, Finland; 14 Turku PET Centre, Turku University Hospital, Turku, Finland

**Keywords:** Obesity, Insulin resistance, Type 2 diabetes, Exercise training, Glucose metabolism, Brain metabolism, Monozygotic twins

## Abstract

**Background:**

Obesity and physical inactivity are major global public health concerns, both of which increase the risk of insulin resistance and type 2 diabetes. Regulation of glucose homeostasis involves cross-talk between the central nervous system, peripheral tissues, and gut microbiota, and is affected by genetics. *Systemic cross-talk between brain, gut, and peripheral tissues in glucose homeostasis: effects of exercise training (CROSSYS)* aims to gain new systems-level understanding of the central metabolism in human body, and how exercise training affects this cross-talk.

**Methods:**

CROSSYS is an exercise training intervention, in which participants are monozygotic twins from pairs discordant for body mass index (BMI) and within a pair at least the other is overweight. Twins are recruited from three population-based longitudinal Finnish twin studies, including twins born in 1983–1987, 1975–1979, and 1945–1958. The participants undergo 6-month-long exercise intervention period, exercising four times a week (including endurance, strength, and high-intensity training). Before and after the exercise intervention, comprehensive measurements are performed in Turku PET Centre, Turku, Finland. The measurements include: two positron emission tomography studies (insulin-stimulated whole-body and tissue-specific glucose uptake and neuroinflammation), magnetic resonance imaging (brain morphology and function, quantification of body fat masses and organ volumes), magnetic resonance spectroscopy (quantification of fat within heart, pancreas, liver and tibialis anterior muscle), echocardiography, skeletal muscle and adipose tissue biopsies, a neuropsychological test battery as well as biosamples from blood, urine and stool. The participants also perform a maximal exercise capacity test and tests of muscular strength.

**Discussion:**

This study addresses the major public health problems related to modern lifestyle, obesity, and physical inactivity. An eminent strength of this project is the possibility to study monozygotic twin pairs that share the genome at the sequence level but are discordant for BMI that is a risk factor for metabolic impairments such as insulin resistance. Thus, this exercise training intervention elucidates the effects of obesity on metabolism and whether regular exercise training is able to reverse obesity-related impairments in metabolism in the absence of the confounding effects of genetic factors.

**Trial registration:**

ClinicalTrials.gov, NCT03730610. Prospectively registered 5 November 2018.

## Background

The increasing prevalence of obesity has become a global public health issue. Obesity and physical inactivity are the causes of life-style-induced diseases such as type 2 diabetes (T2D) [1, 2]. According to the report by World Health Organization (WHO) in 2016, worldwide one in 11 adults has T2D, one in ten is obese, and one in three is overweight. Importantly, most of the world’s population live in countries where obesity is more frequent cause of death compared with undernutrition. Hence, means to fight this global health burden are greatly needed.

Insulin is a vital hormone that is produced in pancreatic beta cells from where it is released into the circulation. Among other functions insulin controls the blood glucose level by promoting the absorption of glucose into the insulin sensitive tissues such as skeletal muscle, liver and fat tissue. Insulin also regulates the amount of glucose that is secreted into the circulation by the liver [3].

Obesity is associated with insulin resistance and low-grade inflammation which induce the impairment in the metabolism and function of peripheral tissues such as liver, skeletal muscle and pancreas [4]. Traditionally, regulation of glucose homeostasis has been considered as an interplay between pancreatic beta cells and peripheral insulin sensitive tissues. Furthermore, the dysfunction of the pancreatic beta cells together with reduced insulin sensitivity of peripheral tissues are believed to be the major cause of the development of insulin resistance and T2D [5]. However, recent advances in neuroscience have provoked an interest to study the role of brain in more detail in obesity and the development of T2D. Interestingly, recent studies have suggested that obesity-induced low-grade inflammation impairs also the brain function and disturbs glucose sensing, insulin signaling and hypothalamic circulation in central nervous system [6–8]. Thus, abnormal brain function induced by obesity may play a role in the development of T2D. Therefore, a question has emerged whether a brain-centric model of glucose homeostasis regulation would better explain the development of insulin resistance compared with the traditional beta cell-centric model in obesity [5, 8].

In addition to the advances in neuroscience, the development of multi-omics sequencing has enabled the study of gut microbiota in greater detail. Intriguingly, the link between the gut microbiota and the development of obesity and insulin resistance is becoming evident [9, 10]. Thus, the current evidence indicates that obesity and insulin resistance are systems-level conditions. However, more in-depth knowledge of the systems-level cross-talk in glucose regulation is needed to understand the pathophysiology and development of T2D in obesity.

The current evidence from family and twin studies clearly indicate that genetics have an important role on the variation of exercise capacity in the sedentary state among healthy people. In addition to the exercise capacity, the response to same exercise protocol is also highly variable between different individuals and explained by genetic factors. In other words, some people improve their aerobic performance to a greater extent than the others, while some people may even worsen their exercise capacity in response to the same exercise training [11, 12]. In addition to family and twin studies, genome wide association studies (GWAS) have also suggested that several single nucleotide polymorphisms may predict the variation how people respond to aerobic exercise training [13].

According to the data acquired from family and twin studies, obesity and metabolic diseases such as T2D, are affected by genetic factors [14–16]. GWAS has suggested that several quantitative trait loci are associated with the development of T2D [17]. Thus, although regular exercise training is a powerful tool to maintain glucose homeostasis and improve insulin sensitivity, individual variation exists in the training response [18–21].

Given that the response to exercise training is heterogeneous and affected by genetic factors, this study utilizes a co-twin control method in order to exclude the confounding effects of genetic factors. To pursue this objective, we recruit monozygotic (MZ) twin pairs that are discordant for body mass index (BMI) (difference ≥ 2 kg•m^− 2^) and at least the other twin is overweight (BMI ≥ 25 kg•m^− 2^). MZ twins share same genes at sequence level, are of the same sex, age and share most of their childhood environment. Thus, they provide an ideal research design to study the effects of regular exercise training on physical performance and metabolic health outcomes, in which the confounding effects of genetic factors can be controlled [22]. Additionally, because twin pairs included into this study are discordant for BMI, it is possible to examine the interaction-effect of different baseline weight and long-term exercise while genetic factors are controlled.

Exercise training provides an effective protection against chronic diseases that are triggered by low-grade systemic inflammation, such as insulin resistance [23]. As a drug-free treatment option, exercise training could lead to significant reduction in treatment cost due to reduced need for medication and fewer complications due to the improved physical fitness. We have previously shown in the Turku PET Centre that only 2 weeks of exercise training has a positive effect not only on the metabolism of the skeletal muscle [24] but also that of the heart [25, 26], the brain [27], and the intestine [28] in healthy, insulin resistant or type 2 diabetic participants. We have also shown that short-time exercise training decreases ectopic fat in the pancreas [29], liver [30], in and around the heart [31] and increases microbial diversity and decreases microbiota content related to obesity and intestinal inflammation [32]. These short-term intervention studies suggest that already a short exercise training period exerts positive systems-level effects on the metabolism of various tissues. Thus, current evidence provided by our research group and others’ [33, 34] suggest that exercise is able to counteract the undesired disturbances in metabolism related to obesity and T2D. However, more specified evidence of the effects of exercise training on different tissues is still needed in overweight/obese people prone to develop metabolic diseases such as T2D.

*Systemic cross-talk between brain, gut, and peripheral tissues in glucose homeostasis: effects of exercise training (CROSSYS)* is an exercise training intervention that provides a unique possibility to examine the systems-level development of insulin resistance without confounding effects of genetic variation. This is because systemic regulation of glucose homeostasis is studied in twins from MZ pairs discordant for BMI and/or insulin resistance. We use state-of-the-art medical imaging technology, positron emission tomography (PET) and magnetic resonance imaging (MRI). All participants will conduct a six-month-long progressive exercise training intervention period that is designed to realistically fit in demanding everyday life. The training program is planned according to the Finnish exercise guidelines and will include endurance, resistance and, high intensity interval training.

## Methods

### Study design

The CROSSYS study is an exercise training intervention with a single group assignment, and the study is prospectively registered on 5th November 2018 in ClinicalTrials.gov with the identifier NCT03730610. We investigate the effects of exercise training on closely related physiological systems that are central for the regulation of the body metabolism. These include: *i.* tissue-specific insulin-stimulated glucose uptake (insulin sensitivity) in brain, liver, intestine, subcutaneous and visceral adipose tissues, in muscle and myocardium, *ii.* whole-body insulin sensitivity, endogenous glucose production and pancreas function, *iii.* Brain morphology and inflammation, *iv.* cognitive functioning, *v.* ectopic fat masses in and around organs, *vi.* adipocyte size, angiogenesis, inflammation, glucose transporters, adipokines and myokines in femoral muscle and abdominal and femoral subcutaneous fat tissues, *vii.* Metabolomics, *viii*. Comprehensive blood profile, *ix.* glucose tolerance and *x*. microbiota diversity and the associations between microbiota and tissue specific insulin sensitivity especially in the gut-brain axis.

**Fig. 1:**
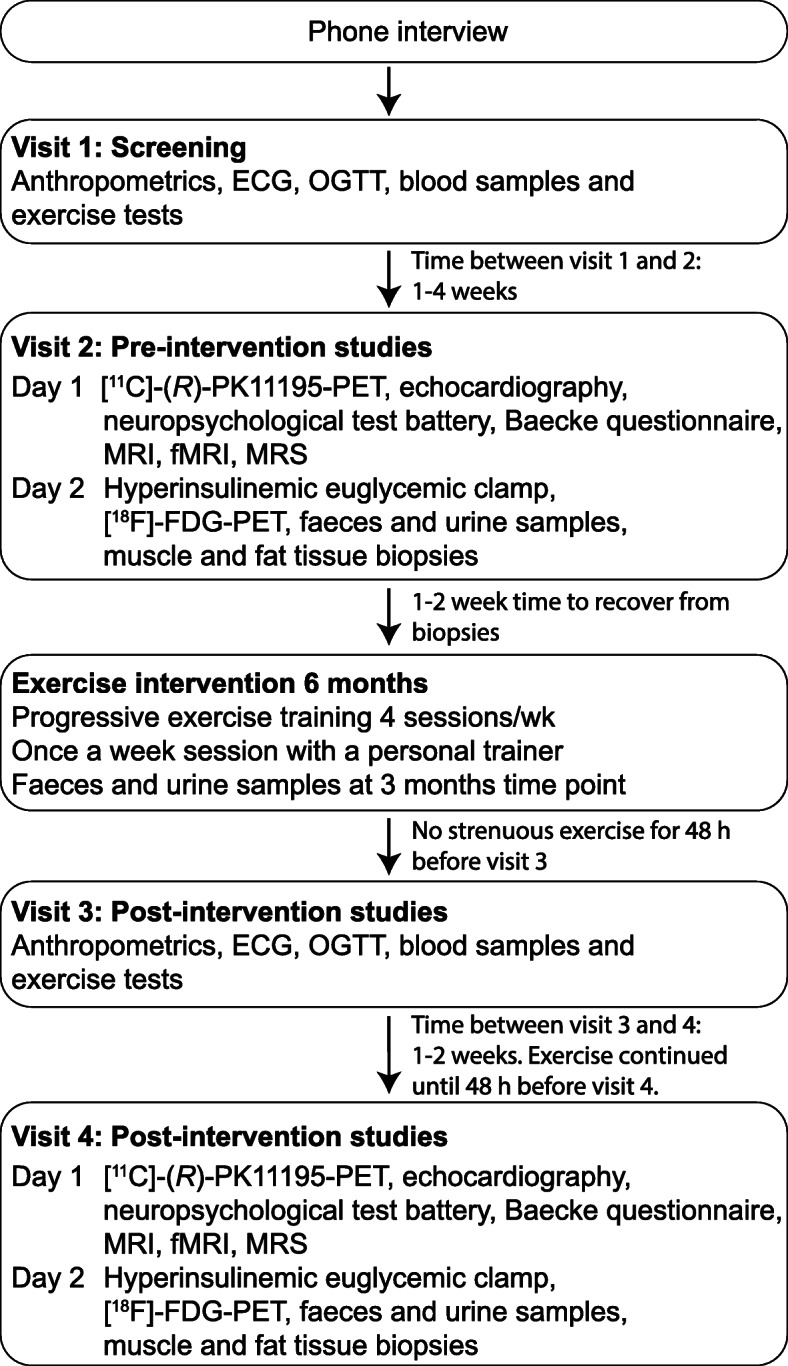
Overview of the study protocol. ECG: electrocardiography; FDG: 2-deoxy-2-[^18^F]fluoro-D-glucose; fMRI: functional magnetic resonance imaging; MRI: magnetic resonance imaging; MRS: magnetic resonance spectroscopy; OGTT: oral glucose tolerance test; [^11^C]-(*R*)-PK11195: ^11^C-labelled R isomer of [1-(2-chlorophenyl)-N-methyl-N-(1-methylpropyl)-3-isoquinolinecarboxamide]; PET: positron emission tomography

The study flow is illustrated in Fig. 1. The eligibility of the participants is first evaluated in a phone interview, followed by a screening visit to ensure that the inclusion criteria for the twin pair are fulfilled. During the screening visit, participants are thoroughly examined by a medical doctor, including electrocardiography (ECG). Within 1–4 weeks after the screening visit, the twin pairs arrive to baseline measurements, which are described in detail below. Then, both co-twins exercise four times a week for 6 months in their place of residence (Table 1), receiving guidance from a personal trainer once a week. At 3 months, participants take a urine and faeces sample and fill out the food diary. After 6 months, the same measurements as at the baseline are repeated (Fig. 1). Participants are asked to avoid strenuous exercise as well as caffeinated drinks and alcohol 48 h before each study visit. The usage of nicotine-products is not restricted prior to the measurements. Participants are required to fast overnight (at least 10 h) before all the study days except for the study day 1 during the visits 2 and 4 (Fig. 1).

**Table 1:** Progressive training intervention for 26 weeks

**ENDURANCE TRAINING (twice a week: one Session 1 and one Session 2)**						
	*Weeks 1–2*	*Weeks 3–4*	*Weeks 5–6*	*Weeks 7–9*	*Weeks 10–19*	*Weeks 20–26*
Session 1	70% HR_max_ 30 min	70% HR_max_ 35 min	70% HR_max_ 40 min	70% HR_max_ 45 min	75% HR_max_ 45 min	80% HR_max_ 45 min
Session 2	60% HR_max_ 40 min	60% HR_max_ 50 min	60% HR_max_ 60 min	60 % HRmax 60 min	60 % HRmax 60 min	60 % HRmax 60 min
**HIGH INTENSITY INTERVAL TRAINING (once a week one type of training)**						
*Type*	*Completion method*			*Content of the training*		
**Circuit**	Perform 4 rounds of the movements in chronological order. Every round, spend 1 min in each movement: do as many repetitions as you can in 40 s and then rest for 20 s. Rest 1 min between the rounds after completing all movements (1–6). **Above 80% of HR**_**max**_			1. Lunges 2. X-jump/jumping rope/mountain climber 3. Back extension 4. Abdominal crunches (obliqual) 5. Burpee 6. Hip thrust 7. Rest		
**Cross training**	First, perform 12 min of section A (switch between two movements). Rest 1 min. Second, perform 6 min of section B. Rest 1 min. Third, perform 6 min of section C. **Above 80% of HR**_**max**_			A. 500-m row/cycling/run 10 air squats B. 20 kettlebell swings 10 push-ups/pull-ups C. 30 sit-ups 30 box step-ups		
**HIIT**	Make each bout as hard as possible. Return between bouts by walking calmly back to the starting point. Repeat 4–6 times. **Above 90% of HR**_**max**_			Choose one: - Running/cycling/rowing - Stair-running - Uphill running		
**RESISTANCE TRAINING (once a week)**						
*Weeks 1–9*	*Weeks 10–19*			*Weeks 20–26*	*Repetitions (load)*	
Leg press	Leg press/back squat			Leg press/back squat	3 × 10, (75% of 1RM)	
Leg extension	Bulgarian squats			Hip extensions	3 × 10, (75% of 1RM)	
Push-ups^a^	Cable seated row			Bent-over row	3 × 10, (75% of 1RM)	
Peck-deck	Bench press			Bench press	3 × 10, (75% of 1RM)	
Lat pulldown	Lat pulldown			Lat pulldown/Pull-ups	3 × 10, (75% of 1RM)	
Shoulder press	Shoulder press			Shoulder press	3 × 10, (75% of 1RM)	
Abdominal crunches^a^	Abdominal crunches^a^			Abdominal crunches^a^	3 × 10	

*HR*_*max*_ maximum heart rate

1 RM: external load that can be lifted once i.e. one repetition maximum

^a^Body weight exercise

The study is conducted at the facilities of Turku PET Centre and Turku University Hospital (Turku, Finland) except for the exercise tests and body composition, which are carried out at Paavo Nurmi Centre (Turku, Finland).

### Participants

**Fig. 2:**
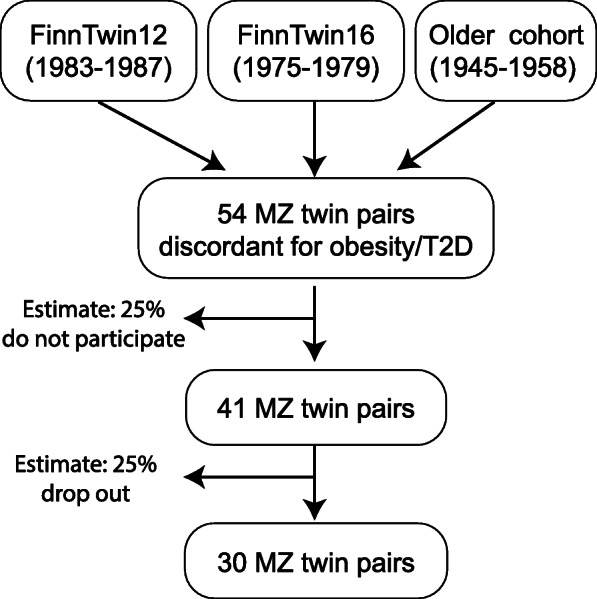
Recruitment of the monozygotic twin pairs from three twin cohorts (FinnTwin12, FinnTwin16 and older cohort) as well as their estimated participation and drop-out rates. MZ: monozygotic, T2D: type 2 diabetes

The participants of the CROSSYS study are monozygotic (MZ) twin pairs that are recruited from three unique population-based longitudinal twin studies [35–37]. Of these three cohorts, the two younger cohorts consist of twins from the FinnTwin12 (FT12, twins born 1983–1987) and FinnTwin16 (FT16, twins born 1975–1979) studies, and twins born in 1945–1957, who form part of the older cohort from the Finnish Twin Cohort Study, twins born in 1945–1957 (Fig. 2). The three cohorts consist altogether of more than 9200 twin pairs, of which 1/3 are MZ. All twins have been identified through Finland’s central population registry, permitting exhaustive and unbiased ascertainment of all twins living and resident in Finland. A total of 54 MZ pairs discordant for BMI and/or insulin resistance have participated previously to the TwinFat study in the University of Helsinki, and their monozygocity has been confirmed by the genotyping of 10 informative genetic markers [38]. All these 54 pairs are invited to participate in the present study. Inclusion criteria are: MZ twins, BMI within-pair difference ≥ 2 kg•m^− 2^ and/or insulin resistance, and at least one of the co-twins is overweight (BMI > 25 kg•m^− 2^). Exclusion criteria are: BMI > 60 kg•m^− 2^, body mass > 170 kg, waist circumference > 150 cm (due to the gantry limitations of PET and MRI machines), mental disorder or poor compliance, eating disorder or excess use of alcohol, active ulcus disease, diabetes requiring insulin treatment or fasting glucose > 10 mmol•l^-1^, pregnancy, past dose of radiation, claustrophobia, presence of ferromagnetic objects that would make MRI contraindicated, physical disability that would prevent exercising, or any other ﻿condition which could potentially endanger participant’s health during the study or interfere with the interpretation of the results. It is estimated that 25% of twins do not want to participate or are not eligible to the study and another 25% will drop out during the intervention. Hence, 30 twin pairs are expected to successfully complete the study.

### Exercise inter**v**ention

#### Contents of the exercise intervention

All study participants perform 26 ± 2-week training intervention period (Table 1). The training period includes one supervised (local personal trainer) and three unsupervised home-based exercise training sessions per week. The mixed-type intervention period consists of endurance, high intensity interval and resistance training. The intensity of the training is progressive, and the individual training loads are determined with the help of a personal trainer to meet the intensities of the protocol (Table 1). The training intervention was planned by three exercise physiologists (M.A.H., S.M.H, J.C.H.). Training program includes two deload weeks in order to avoid overtraining and injuries. These deload periods are weeks 9 and 18. During the deload weeks, participants perform only endurance training.

Principally, all the subjects exercise according to the planned flexible training program. However, small adjustments to the exercises can be individually modified to achieve a better adherence to the planned exercise intervention. For instance, if the participant has musculoskeletal system disabilities that preclude planned exercises, these are replaced with a more suitable one that differs from the original as little as possible. The modifications are suggested by the personal trainer and approved by the researchers.

##### Endurance training

Endurance training can be implemented in many ways, such as running, cycling, swimming or rowing. The participant is allowed to choose the most suitable forms of exercise and the participant is free to switch between different forms of training. Endurance training is performed twice a week and monitored by a heart-rate monitor (Polar A370, Polar, Finland). The duration and the intensity of the training increases progressively (Table 1).

##### High Intensity Interval Training (HIIT)

High intensity interval training (HIIT) consisting of circuit type training, cross-training and uphill−/stair-running is performed once a week. HIIT includes three different exercise sessions that change every 2 weeks (Table 1.). The idea in HIIT is to perform short exercise bouts with very high intensity with recovery periods in between.

##### Resistance training

Resistance training (RT) is performed once a week and the loads correspond to approximately 75% of the external load that can be lifted once i.e. one repetition maximum (1 RM) throughout the training period. The training program includes both upper and lower body exercises at each session (Table 1). The initial training loads for different training phases and specific exercises are determined at weeks 1, 10 and 20. The exercises become more challenging as the intervention progresses, if the participant’s development and body control allows.

The adherence of the participants to the exercise interventions are monitored using heart rate monitors (Polar A370, Polar, Finland). The participants are asked to upload the heart rate monitor data into the PolarFlow cloud service periodically. In addition, participants are provided with an exercise diary to record all completed exercises and their dates. Missed exercises and their causes are also recorded in the diary. Based on the exercise diary and heart rate monitor data, the researchers assess whether a sufficient proportion of the exercises have been completed. The study participants are not allowed to participate in another concurrent interventional clinical trial during the study period.

#### Personal training

Study participants live in different parts of Finland, and therefore the exercise intervention is performed at the participants’ local gym and cannot be supervised by the CROSSYS research group per se. The gyms are chosen according to the participants’ preference as well as evaluation of the gym facilities. The selection criteria for gyms includes comprehensive gym equipment and personal trainer services, as the study’s personal trainers are recruited through selected gyms. Personal trainers are introduced to research and exercise intervention on a phone conversation and are given written instructions regarding the training period. In addition, they receive the necessary information regarding the participants’ background and possible limitations that need to be taken into account when planning the individually tailored exercise program. Personal trainers’ main role is to motivate the participants to stick with the planned exercise intervention and to supervise the participants to conduct the exercises as they were planned with a proper technique to avoid injuries. Personal trainers are also advised on what to do, for example, if the participant becomes ill or has to cancel training. Personal trainers are also advised to contact the research team if anything unexpected or abnormal happens.

### Outcome measures

#### Euglycemic hyperinsulinemic clamp and FDG-PET study

Tissue-specific insulin-stimulated glucose uptake is one of the primary outcome measures in this study. It is studied with a radio tracer 2-deoxy-2-[^18^F]fluoro-D-glucose (FDG) by PET. FDG is a glucose analogue that is transported into cells similarly as glucose. In a cell, FDG is phosphorylated, which prevents its further metabolism and it is therefore trapped within the cell until it decays and emits photons that are detected by the PET scanner [39]. In the present study FDG-PET scan is performed during an euglycemic hyperinsulinemic clamp [40] that is a method to measure whole-body insulin-stimulated glucose uptake (M-value). This value is considered to be the most definitive measure of insulin sensitivity in humans and is another primary outcome measure of this study [41].

Participant is required to fast overnight (at least 10 h) and to avoid excess physical activity for 48 h before the FDG-PET study. Before the euglycemic hyperinsulinemic clamp and the PET study protocol, the antecubital veins from both arms are cannulated. One of the two catheters is used for the administration of glucose and insulin during the clamp study and injection of the PET tracer. The other catheter is used to obtain venous blood samples during the study. The arm used to obtain blood samples is heated with an electrically powered cushion for the whole duration of the study to “arterialize” the venous blood. The participant is positioned in a supine position and instructed not to move and avoid muscle contraction throughout the whole study protocol.

The euglycemic hyperinsulinemic clamp is performed as originally described by DeFronzo et al [41]. A primed-constant insulin (Actrapid 100 U · ml^− 1^, NovoNordisk, Bagsvaerd, Denmark) infusion is started with the rate of 40 mU per square meter of body surface area in minute (mU·m^− 2^·min^− 1^) during the first 4 min. Then, the infusion rate is reduced to 20 mU·m^− 2^·min^− 1^ for the time interval 4–7 min. After 7 min, the infusion rate is further reduced to 10 mU·m^− 2^·min^− 1^ for the rest of the clamp. Exogenous glucose infusion is started 4 min after the start of the insulin infusion with a rate of [participant’s mass (kg)·0.5]g·h^− 1^. At the 10-min time point, glucose infusion is doubled and after that further adjusted according to blood glucose concentration aiming at the steady level of 5 mmol·l^− 1^. Arterialized venous blood samples are collected before the clamp and every 5 min during the entire clamp to determine the glucose concentration for adjusting the glucose infusion rate. M-value is calculated from the glucose values obtained in the steady state of 5 mmol·l^− 1^ lasting at least 20 min [41].

After 60 min of insulin clamp, a participant is positioned in a supine position into the PET scanner (Discovery MI (DMI), GE Healthcare, US), while all unnecessary movement and muscle activity of the participant is avoided during the transition. Tissue-specific insulin-stimulated glucose uptake is measured using FDG as a radio tracer, which is produced according to Hamacher et al. [42]. The tracer [150 MBq] is injected to the antecubital vein via a catheter approximately 80–90 min after the start of the euglycemic hyperinsulinemic clamp when the stable glucose concentration of 5 mmol∙l^− 1^ (±0.5) is reached. Once the tracer is injected to the participant, scanning is started immediately. The scanning protocol starts from brain and lasts for 40 min in 4 × 30 s, 3 × 60 s, and 7 × 300 s time frames. Then, the scanning proceeds to thoracic, abdominal and femoral regions for 9 min in 3 × 180 s time frames for each of these three regions. Blood samples for plasma radioactivity determination (Wizard 1480 Automatic Gamma Counter, Perkin-Elmer) and calculation of input function are collected at 30, 45, 60, 75, 90 s, and 2, 2.5, 3, 3.5, 4, 4.5, 5, 10, 15, 20, 30, 35 min after the injection of the tracer during the brain scan and once at 4.5 min during each of the consecutive three 9-min scans (thoracic, abdominal and femoral region scans). Euglycemic hyperinsulinemic clamp is continued throughout the FDG-PET scan, and blood glucose levels are monitored by taking blood samples every 5 min.

During the FDG-PET study, blood samples for lactate are collected at time points 0, 20, and 80 min, for insulin at 0, 30, 60, 90, 120, and 150 min, and for free fatty acids at 0, 60 and 120 min. These samples are collected to ensure that the clamp is performed successfully.

All obtained PET image raw files are corrected for attenuation, dead time, and decay. Images are reconstructed using the block sequential regularized expectation maximization (BSREM) algorithm with BETA factor 150 for the brain and 350 for thoracic, abdominal and femoral regions. Images are analysed using Carimas software that has been produced *in house* (www.turkupetcentre.fi/carimas).

#### PK11195-PET

Neuroinflammation is another outcome measure in this study and studied using PET imaging with a ^11^C-labelled R isomer of [1-(2-chlorophenyl)-N-methyl-N-(1-methylpropyl)-3-isoquinolinecarboxamide] ([^11^C]-(*R*)-PK11195), which binds to the 18-kDA translocator protein (TSOP) [43]. TSOP is upregulated in activated microglial cells and macrophages associated with neuroinflammation, but is expressed at low level in resting microglia [44]. Thus, [^11^C]-(*R*)-PK11195 can be used as a marker of inflammation specifically in brain.

PK11195-PET study is performed after a light meal. Before the PK11195-PET study, antecubital vein of the left hand is cannulated and fed-state blood samples for C-reactive protein and microglial cell quantification is taken in order to study possible correlations between these inflammation markers and PK11195-PET imaging results.

The participant is then positioned into the PET scanner (Discovery MI (DMI), GE Healthcare, US), in supine position with the brain in the scanning area of the gantry. The PET/computed tomography (CT) scanner used in the PK11195-PET study is the same scanner used in the FDG-PET study. Once the tracer of 350 MBq is injected into the vein, the scanning is started immediately and continued for 60 min in 2 × 15 s, 3 × 30 s, 3 × 60 s, 7 × 300 s, and 2 × 600 s time frames. Blood samples are not taken during the PK11195-PET scan. Images are reconstructed using the BSREM algorithm with BETA factor 350.

#### Magnetic resonance imaging and magnetic resonance spectroscopy

Magnetic resonance imaging (MRI) and proton magnetic resonance spectroscopy (MRS) studies are performed in the evening approximately four h after the lunch. Participants have a small snack before the study. The imaging is performed with Siemens Magnetom Skyra fit 3 T MRI system (Siemens Healthcare, Erlangen, Germany). MRI is performed in five parts as detailed below. Participants are allowed to have a break between the parts when necessary.

At first, the brain MRI is performed with a Siemens Head/Neck 20 channel coil, including brain morphology and the resting state functional MRI (fMRI) sequences. The brain protocol consists of six sequences as listed in Table 2 (localizer not listed). The resting state fMRI with eyes open is acquired twice.

**Table 2:** Parameters of brain MRI sequences

Sequence	Slice orientation	Phase encoding direction	Number of slices	Field of view (mm^2^)	Slice gap (mm)	Voxel size (mm^3^)	Repetition time (ms)	Echo time (ms)	Inversion time	Flip angle (°)	Bandwidth (Hz/Px)	Imaging mode	Acceleration mode (factor)
T2 TSE	Transversal	R> > L	35	224 × 182	0.8	0.6 × 0.6 × 4.0	6600.0	89.0	N/A	150	224	2D	GRAPPA (2)
T1 MPRAGE	Sagittal	A> > P	192	256 × 240	N/A	1.0 × 1.0 × 1.0	2300	2.26	900	8	200	3D	GRAPPA (2)
FLAIR	Sagittal	A> > P	192	256 × 240	0	1.0 × 1.0 × 1.0	5000	386	1800	Variable	751	3D	GRAPPA (3)
Resting-state fMRI	Transversal	A> > P	48	220 × 220	0	3.4 × 3.4 × 3.4	3000	30	N/A	90	1562	2D	GRAPPA (2)
Field mapping	Transversal	R> > L	48	220 × 220	0	3.4 × 3.4 × 3.4	350	4.92; 7.38	N/A	60	290	2D	N/A

*TSE* Turbo Spin Echo, *GRAPPA* GeneRalized Autocalibrating Partial Parallel Acquisition, *MPRAGE* Magnetization Prepared RApid Gradient Echo, *FLAIR* Fluid-Attenuated Inversion Recovery, *fMRI* Functional Magnetic Resonance Imaging, *CAIPIRINHA* Controlled Aliasing in Parallel Imaging Results in Higher Acceleration, *HASTE* Half-Fourier Acquisition Single-shot Turbo spin Echo imaging, *VIBE* Volometric Interpolated Breath-hold Examination, *TRUFI* TRUe Fast Imaging with steady-state free precession

Secondly, a whole-body imaging is performed in order to define volume of adipose tissue in abdominal and femoral regions as well as volume of the liver and the pancreas. At first, a rapid whole-body localizer image is acquired using a FastView sequence utilizing a continuously moving table (table speed 46 mm·s^− 1^). Then, T1 VIBE Dixon images are acquired in five substeps which are 200 mm apart from each other (center to center). The general parameters of the series after the localizer sequences are listed in Table 3. A combination of Spine 32, Head/Neck 20, Body 30, Body 18 and Peripheral Angio 36 coil is used for signal acquisition.

**Table 3:** Parameters of whole-body MRI sequences

Sequence	Slice orientation	Phase encoding direction	Number of slices	Field of view (mm^2^)	Slice gap (mm)	Voxel size (mm^3^)	Repetition time (ms)	Echo time (ms)	Respiration control (parts)	Flip angle (°)	Bandwidth (Hz/Px)	Imaging mode	Acceleration mode (factor)
FastView	Transversal	A> > P	1	480 × 420	5	5 × 5 × 5	2.56	1.44	none	N/A	801.3	2D	N/A
T1 VIBE Dixon	Transversal	A> > P	120	448 × 364	N/A	2 × 2 × 2	3.97	1.23; 2.46	Breath-hold (1)	9	1060	3D	CAIPIRINHA (4)

*VIBE* Volometric Interpolated Breath-hold Examination, *CAIPIRINHA* Controlled Aliasing in Parallel Imaging Results in Higher Acceleration

The third object is the heart, where pericardial fat volume is determined as well as the fat content within the septum by using MRS. The heart MRI protocol consists of 9 sequences as detailed in Table 4 (localizer sequences not listed) using a combination of Siemens Body 30 and Spine 32 coils. The first five sequences are used for positioning and planning of the two cine sequences to acquire them in four-chamber and short-axis orientations. T2 Haste sequence is used for positioning the breathing navigator voxel for the T1 turbo spin echo (TSE) dark blood sequence which is acquired for the analysis of pericardial fat volume. All sequences are acquired under breath holds. All but the T2 Haste and localizer are ECG triggered. In the cine sequences 30 frames were acquired in one ECG R-R interval.

**Table 4:** Parameters of heart MRI sequences

Sequence	Slice orientation	Phase encoding direction	Number of slices	Field of view (mm^2^)	Slice gap (mm)	Voxel size (mm^3^)	Repetition time (ms)	Echo time (ms)	Respiration control (parts)	Flip angle (°)	Bandwidth (Hz/Px)	Imaging mode	Acceleration mode (factor)
TRUFI single shot axial	transversal	A> > P	10	340 × 252	6.0	1.3 × 1.3 × 6.0	295.68	1.23	Breath-hold (1)	38	849	2D	GRAPPA (2)
TRUFI single shot 2 chamber	2-chamber	L> > R	1	340 × 252	1.5	1.3 × 1.3 × 6.0	295.68	1.23	Breath-hold (1)	38	849	2D	GRAPPA (2)
TRUFI single shot nearly 4 chamber	nearly 4-chamber	A> > P	1	340 × 252	1.5	1.3 × 1.3 × 6.0	295.68	1.23	Breath-hold (1)	38	849	2D	GRAPPA (2)
TRUFI single shot short axis	short-axis	A> > P	1	340 × 252	1.5	1.3 × 1.3 × 6.0	295.68	1.23	Breath-hold (1)	38	849	2D	GRAPPA (2)
TRUFI single shot 4 chamber	4-chamber	A> > P	1	340 × 252	1.5	1.3 × 1.3 × 6.0	295.68	1.23	Breath-hold (1)	38	849	2D	GRAPPA (2)
Cine 4 chamber	4-chamber	A> > P	4	340 × 276	1.2	1.8 × 1.8 × 6.0	35.70	2.47	Breath-hold (4)	12	449	2D	GRAPPA (2)
Cine short axis	short-axis	A> > P	6	340 × 276	1.2	1.8 × 1.8 × 6.0	35.70	2.47	Breath-hold (6)	12	449	2D	GRAPPA (2)
T2 HASTE	Coronal	R> > L	30	400 × 400	1.0	1.6 × 1.6 × 5.0	1400	87	Breath-hold (2)	160	698	2D	GRAPPA (3)
T1 TSE dark blood	transversal	A> > P	20	340 × 276	2.0	1.3 × 1.3 × 4.0	750	4.1	Breath-hold (20)	180	849	2D	GRAPPA (3)

*TSE* Turbo Spin Echo, *GRAPPA* GeneRalized Autocalibrating Partial Parallel Acquisition, *HASTE* Half-Fourier Acquisition Single-shot Turbo spin Echo imaging, *VIBE* Volometric Interpolated Breath-hold Examination, *TRUFI* TRUe Fast Imaging with steady-state free precession

The fourth imaging region is the abdomen (Table 5, localizer sequences not listed), in which the fat content within the pancreas and the liver is determined by MRS using Spine 32, Siemens Body 30 and Siemens Body 18 coils. Finally, the fifth object is the tibialis anterior muscle measured with QED TxRx 15 channel knee coil (Table 6, localizer sequences not listed), in which intra- and extramyocellular lipid content is measured by MRS. Two MRS spectra are acquired from each organ (heart, pancreas, liver, tibialis anterior): one with water suppression and another without water suppression. For the heart, delay for the ECG trigger is adjusted for each participant, typical value being 210 ms. The general sequence parameters for MRS are listed in Table 7.

**Table 5:** Parameters of liver and pancreas MRI sequences

Sequence	Slice orientation	Phase encoding direction	Number of slices	Field of view (mm^2^)	Slice gap (mm)	Voxel size (mm^3^)	Repetition time (ms)	Echo time (ms)	Respiration control	Flip angle (°)	Bandwidth (Hz/Px)	Imaging mode	Acceleration mode (factor)
T2 HASTE	Transversal	A> > P	49	380 × 309	1	0.7 × 0.7 × 5.0	1570.0	75; 420	Navigator	114	977	2D	N/A
T2 HASTE	Sagittal	A> > P	40	380 × 285	1	1.2 × 1.2 × 5.0	500.0	95	Navigator	160	710	2D	GRAPPA (2)
T2 HASTE	Coronal	R> > L	30	450 × 394	1	1.4 × 1.4 × 5.0	656.0	95	Navigator	160	710	2D	GRAPPA (2)

*HASTE* Half-Fourier Acquisition Single-shot Turbo spin Echo imaging, *GRAPPA* GeneRalized Autocalibrating Partial Parallel Acquisition

**Table 6:** Parameters of the tibilais anterior muscle MRI sequences

Sequence	Slice orientation	Phase encoding direction	Number of slices	Field of view (mm^2^)	Voxel size (mm^3^)	Repetition time (ms)	Echo time (ms)	Flip angle (°)	Bandwidth (Hz/Px)	Imaging mode	Acceleration mode (factor)
T1 VIBE Dixon	Transversal	A> > P	144	268 × 201	1.4 × 1.4 × 1.0	4.14	1.23; 2.46	9.0	1040	3D	CAIPIRINHA (4)
T1 VIBE in-opp	Transversal	A> > P	160	192 × 156	1.0 × 1.0 × 1.0	4.76	1.51; 3.01	9.0	870	3D	None
T1 VIBE in-opp	Sagittal	A> > P	160	256 × 152	1.0 × 1.0 × 1.0	4.76	1.51; 3.01	9.0	850	3D	None
T1 VIBE in-opp	Coronal	R> > L	104	256 × 152	1.0 × 1.0 × 1.0	4.78	1.51; 3.01	9.0	850	3D	None

*VIBE* Volometric Interpolated Breath-hold Examination, *CAIPIRINHA* Controlled Aliasing in Parallel Imaging Results in Higher Acceleration

**Table 7:** Parameters of the 1H MRS sequences in the heart (septum), pancreas, liver and tibialis anterior muscle

Sequence	Sequence type	Voxel size (mm^3^)	TE (ms)	TR (ms)	Number of signal averages	Bandwidth (Hz)	Number of datapoints	Water suppression	Water suppression bandwidth	Heartbeat control	Respiration control
Heart water-suppressed	PRESS	10 × 10 × 10	33	2000	128	2500	1024	Water saturation	35	ECG trigger	Navigator
Heart not water-suppressed	PRESS	10 × 10 × 10	33	2000	16	2500	1024	None	35	ECG trigger	Navigator
Pancreas water-suppressed	PRESS	15 × 15 × 10	30	4000	64	2500	1024	Water saturation	35	None	Navigator
Pancreas not water-suppressed	PRESS	15 × 15 × 10	30	4000	16	2500	1024	None	35	None	Navigator
Liver water-suppressed	PRESS	20 × 20 × 20	30	4000	32	2500	1024	Water saturation	35	None	Navigator
Liver not water-suppressed	PRESS	20 × 20 × 20	30	4000	4	2500	1024	None	35	None	Navigator
Tibialis water-suppressed	PRESS	12 × 12 × 20	33	4000	64	2500	1024	Water saturation	35	None	None
Tibialis not water-suppressed	PRESS	12 × 12 × 20	33	4000	16	2500	1024	None	35	None	None

#### Transthoracic echocardiography

Complete two-dimensional (2D), color, pulsed and continuous-wave Doppler echocardiogram is performed according to standard techniques. Conventional left-ventricular (LV) systolic function is measured with 2- and 4-chamber apical views (Simpson’s biplane method of discs, corrected for body surface area). LV end-diastolic volume index (EDVI), LV end-systolic volume index (ESVI) and LV ejection fraction (LVEF) are calculated. LV mass index is calculated using the area-length method as recommended by the American Society of Echocardiography and corrected for body surface area [45]. Advanced LV systolic function parameters are measured with tissue Doppler imaging (TDI). Peak systolic tissue velocities are derived from septal and lateral pulsed wave and color coded TDI. Speckle tracking derived strain parameters are obtained from apical 4,2 and 3-chamber views [46].

Conventional LV diastolic function parameters are derived from 2- and 4-chamber views [47]. Maximum left atrium (LA) volume is derived with discs method, corrected for body surface area. Pulsed wave Doppler velocities are measured from the apical 4-chamber view using a 2 mm sample volume positioned at mitral leaflet tips. Transmitral early (E wave) and late (A wave) diastolic velocities are measured as well as E wave deceleration time. Pulsed wave TDI of the lateral and septal mitral annulus are used to measure E’.

#### Bone density

Quantitative computed tomography (QCT) is used to measure bone mineral density using Discovery MI computed tomography (CT) scanner (GE Healthcare, Waukesha, WI, US) with a solid Mindways QCT Phantom (Mindways Software Inc., Austin, TX, US, model QCT Pro) immediately after the FDG-PET scan [48]. Vertebrae L2 to L4 are scanned in the supine position. Calibration phantom is used to convert Hounsfield Units into bone mineral equivalents in mg · cm^− 3^ using QCT Pro software (Mindways Software Inc., Austin, TX, USA), with which the CT images are analyzed [49].

#### Blood samples

During the visits 1 and 3 (Fig. 1), blood samples are obtained in a fasted state (10 h) in the morning. From the samples complete blood count, glycated hemoglobin (HbA1c), lipid profiles, liver enzymes and C-reactive protein are determined. Before the FDG-PET study (visits 2 and 4 in Fig. 1), fasting state blood samples are collected and stored in a freezer (− 70 °C) for further analyses.

#### Oral glucose tolerance test

A 2-h oral glucose tolerance test (OGTT) is performed after at least a 10-h fast during the visits 1 and 3. (Fig. 1). After ingestion of 250 ml of liquid containing 75 g of glucose (﻿GlucosePro, Comed Oy, Ylöjärvi, Finland), blood samples are collected at baseline, 15, 30, 45, 60, 90, and 120 min during the test to determine glucose, C-peptide, and insulin concentrations in the blood.

#### Muscle and adipose tissue samples

Muscle and adipose tissue biopsies are collected at the end of the PET imaging visit in a post-prandial state. Subcutaneous adipose tissue is collected from the right side of the lower abdomen and the lateral side of the right thigh using open surgical procedure. After the selected skin area is sterilized and local anesthetic (lidocaine supplemented with epinephrine) administered, a small incision is made from which the adipose tissue sample is extracted with a sharp knife and anatomical forceps. Muscle biopsy is collected from the vastus lateralis muscle inferior to trochanter major and anterior to lateral fascia using suction-modified Bergström muscle biopsy technique [50] from the same cut as the femoral adipose tissue sample. Post-intervention tissue samples are collected as near to the locations of the baseline samples as possible, avoiding the scars. One part of the biopsies is flash frozen in liquid nitrogen and stored at freezer (− 70 °C). The other part is put in formalin and subsequently embedded in paraffin. The incisions are closed with absorbable sutures.

#### Faeces and urine samples

Faeces and urine samples are collected at the first PET imaging visit to determine gut microbiota and metabolomics, respectively, midway through the training intervention, and at the last PET imaging visit. The participants fill out a 3–4 day food diary before giving the faeces sample. The samples are stored in a freezer (− 70 °C) prior to a total DNA extraction and further analysis.

Total DNA will be extracted from the fecal material (approx. 100–200 mg) using a commercial DNA extraction Kit following the manufacturer’s instructions. DNA concentration will be measured using Qubit® 2.0 Fluorometer (Life Technology, Carlsbad, CA, US) for further analysis.

DNA libraries will be performed with the amplification of the Specific 16S ribosomal RNA (rRNA) gene region (V3-V4) following Illumina protocols. A multiplexing step will be conducted by the NextEra XT Index Kit (Illumina, San Diego, CA, United States). 16S amplicons will be confirmed and checked with a Bioanalyzer DNA 1000 chip (Agilent Technologies, Santa Clara, CA, United States). Libraries will be sequenced on Illumina platform according to manufacturer instructions. During DNA extraction and PCR amplification, the reagent contamination is controlled and ruled out by using blanks not containing a sample.

Quality-trimmed and filtering will be assessed using DADA2 pipeline. Taxonomic assignment will be conducted using the Silva v132 database. Samples with less than 1000 reads will be removed from the final analysis. Sequences not assigned to Bacteria domain level, also, those sequences classified as cyanobacteria and chloroplasts, likely represent ingested plant material, will be removed from the dataset.

#### Neuropsychological test battery

Neuropsychological function is assessed by an online survey using Gorilla Experiment Builder (gorilla.sc) platform [51]. The survey consists of several tasks that measure working memory (N-back tasks with *N* = 1 and *N* = 2, digit span text entry), memory encoding and retrieval with CERAD Word List Memory task type test, vigilance, simple reaction time, and fluid intelligence with The matrix reasoning item bank (MaRs-IB) [52] which is a modified open-source variant of the Raven’s progressive matrices test. Emotional sensitivity was measured by asking subjects to report their feelings of valence (pleasure-displeasure) and arousal to a set of pleasant, unpleasant and neutral pictures derived from the International Affective Picture system (IAPS) [53, 54]. The survey is performed after the lunch during an afternoon of a PET study day in a quiet room using a standard desktop computer.

#### Physical activity questionnaire

Habitual physical activity is assessed using a questionnaire by Baecke et al. [55] before and after the exercise intervention period. It consists of 16 questions divided into three sections: physical activity at work, during leisure time (sport excluded) and during sport. Three indices measuring the level of habitual physical activity are calculated (work, leisure and sport index). The questionnaire is performed as an in-person interview conducted by a researcher during a PET study day.

#### Body composition, maximal exercise capacity and muscular strength

Body composition measurements, a maximal oxygen uptake (VO_2max_) exercise test and tests for muscular strength are performed to each participant before and after the training intervention. The tests are performed at the Paavo Nurmi Centre (Turku, Finland).

Participants’ body composition is measured using a bioimpedance analysis machine (Inbody 720; Biospace Co, Korea). The machine measures participants’ total mass, skeletal muscle mass, body fat mass, body mass index, body fat percentage, and the lean mass balance between left and right arm, left and right leg and the trunk.

VO_2max_ is measured using a stationary bicycle ergometer (Ergoline 800 s; VIASYS Healthcare, Germany). For men, the test starts with 50 W which is increased by 30 W every 2 min until volitional exhaustion. For women, the test starts with 40 W which is increased by 20 W every 2 min until volitional exhaustion. Ventilation and gas exchange (Jaeger Oxycon Pro; VIASYS Healthcare or Vyntus CPX, Vyaire Medical Gmbh, Leibnizstrasse, Hoechberg, Germany) are measured during the test. The PRE and POST test for each pair is conducted using the same device. Participants’ blood lactate concentration is measured from capillary samples immediately and 1 min after exhaustion (YSI 2300 Stat Plus; YSI Incorporated Life Sciences, Yellow Springs, OH). The heart rate of the participants is followed continuously with an ECG-machine (CardioSoft GE, CardioSoft V6.51; GE Medical Systems Information Technologies, USA).

Muscle strength is tested using repetition tests for sit-ups, back extensions, and dumbbell overhead press. Maximal grip strength of the dominant hand and indirect test for vertical jump height are also performed. For abdominal muscle strength, participants perform as many repetitions of abdominal crunches as possible in a 30 s time frame. The participants lie on their back in an ab bench with legs fixed into a 90° knee angle, hold hands behind the neck and point elbows forward. Participants’ elbows and knees must touch every repetition. The back strength is measured with a Roman chair with added metal rods that form a 90° angle. Participants perform back extensions with hands behind the neck and elbows pointing directly to the sides as many repetitions as possible in a 30 s time frame. During one repetition participants’ elbows must hit both the upper and the lower metal rods. Arm strength is measured with a standing dumbbell overhead press test. The mass of the dumbbells used during the test are 10 kg for men and 5 kg for women. The aim of the test is to get as many repetitions as possible alternating arms every repetition. Participants are allowed to use only their stronger arm in the end of the test, if a failure in the concentric phase for the weaker arm occurs, in order to conduct as many repetitions as possible. Once the participant has chosen to use only one arm, he or she cannot use the other arm during the test. Maximal grip strength is measured with a computerized digital dynamometer (GOOD STRENGTH dynamometer Metitur Oy, Jyväskylä, Finland). Participants may choose which arm they use for the test. Explosive leg muscle strength is assessed from a vertical counter-movement jump height that is indirectly calculated from the flight time that is recorded by a led-light beam system (OptoJump Next; Microgate, Italy). Participants are instructed to hold their hands at the waist to nullify the effect of the arms during a countermovement-jump. Participants jump 3–5 times and the average of the three best flight times is used to calculate the jump height.

### Statistical analysis and modelling

#### Power calculation

The sample size calculations are based on liver fat content and VO_2max_ results from the earlier cross-sectional study in twins discordant for physical activity and fitness [56, 57], and on M-value [58]. The data will be analyzed with hierarchical linear mixed model which take into account the correlation between repeated measurements and twins. Model will at least include training as categorical factor and time as within factor. It will be investigated whether there are significant change in mean values over time (training effect) and whether training effect is different between the heavier and the leaner twins.

For the VO_2max_, to test the effectiveness of the intervention, the evaluated sample size is calculated using the mean VO_2max_ value for the less active twins [57]. Standard deviation (SD) between twin pair measurements was 2.8 and correlation between measurements over 0.93. With these assumptions we wanted to evaluate the sample size needed to detect a 5–10% mean difference. Due to high correlation, the sample size needed for a 5% mean difference is 6 twin pairs. For a greater mean difference than 5%, the sample size would be even lower. The corresponding values for liver fat content are 2.1%, SD 1.39, and correlation over 0.98 and due to high correlation, the sample size needed for a 20% mean difference is 22 twin pairs [56]. These values for the whole-body insulin sensitivity are 6.2 mg/kg fat-free mass/min, SD 0.3, and correlation over 0.7, and the sample size needed to detect 30% mean difference is 6 twin pairs [58].

According to these calculations, at least 22 pairs is needed for the study. We are unable to calculate the sample size for all the main parameters due to non-existing twin data. However, we believe that the same schema will hold true in the other main parameters due to the high correlation of twins and small variation within the twin pairs.

#### Statistical analysis

The data is first evaluated visually based on standard techniques including quantile-quantile plots and histograms to detect possible outliers and to assess the normality. Normality is also evaluated using Shapiro-Wilk test and logarithm or other statistical transformations are performed when appropriate. The data is also analyzed for possible dimensionality reduction using standard methods such as principal component analysis.

Statistical analyses are performed using hierarchical mixed linear models. The models include two within-factors: time (before and after intervention) and twin (heavier and leaner co-twin), and their interaction term (time x twin). Medications, adherence to the exercise intervention, sex, age, BMI and other factors are included in the models when appropriate. The potential covariates will be screened for association, and covariates that do not show significant association with the response variable will be excluded from the analysis. When the number of participants allows it, we will carry out separate subgroup analyses by sex, age (two younger cohorts vs. older cohort), and/or mass (obese vs. non-obese participants). Subjects with missing data points are included in the statistical analysis by using the restricted maximum likelihood estimation within the linear mixed models. Hence, model-based means and 95% confidence intervals are reported.

Correlation analysis are performed using Pearson’s product-moment correlation coefficient for normally distributed data and Spearman’s rank correlation coefficient for non-normally distributed data. All statistical tests are performed as two-sided and *p*-values less than 0.05 will be considered as statistically significant.

## Discussion

This study allows the investigation of the brain, peripheral tissues, and gut microbiota using sophisticated biomedical imaging technology, clinical and physiological measures combined with multiple omics. The study will reveal new knowledge of the systemic pathophysiological effects of obesity, insulin resistance and T2D and investigates, whether exercise training can alleviate or even reverse these pathophysiological changes.

The benefits of exercise training and the effects of physical inactivity on the whole-body metabolism and health are generally and widely accepted [59, 60]. However, the health benefits of exercise training are often studied using epidemiological studies or cross-sectional studies comparing persons with varying levels of physical activity. The participants in previous studies range from sedentary participants to even elite athletes, while well-planned long-term interventional studies are not very common. In previous cross-sectional studies of MZ pairs discordant for exercise, we see differences in cardiorespiratory fitness [57, 61], body adiposity [56, 61, 62], metabolic parameters [63, 64] and diabetes risk [65, 66] but not in overall mortality [67]. These observational studies permit demonstration of associations, but do not provide strong evidence for causality. Studies with exercise interventions are generally quite short-term with genetically controlled interventional studies being rare. Nonetheless, given genetic influences on both responses to exercise and metabolic outcomes [11, 19], confounding by genetic factors is a major potential source of bias. In the CROSSYS study, the exercise training consists of 6-month-long progressive program that is designed to be realistic in every-day life and is consistent with the current Finnish exercise recommendations. Hence, the exercise program used in this study can be translated into the real-life settings.

The eminent strength of the study is the possibility to study MZ twin pairs discordant for BMI. The co-twin-pairs share a similar genome at sequence level that enables us to study the effects of obesity and insulin resistance in conjunction with long-term exercise training without the confounding effects of genetic variation. All of the twins to be recruited in CROSSYS have participated in previous twin studies in the University of Helsinki. This enables us to use longitudinal data on the development of the obesity and the changes in habitual physical activity of the twins [38, 68]. Another great strength of the present study is the use of state-of-the-art medical imaging technologies, which makes it possible to study tissue-specific metabolism (brain, heart, liver, pancreas, intestine, bone, adipose and muscle tissues) and brain function in detail.

The potential limitation of the study is that the observed changes may reflect changes in the diet. In the present study, we aim to investigate the effects of exercise, and we ask the participants not to change their diet during the 6-month-long exercise intervention. We also advice the personal trainers to help the participants to maintain their diet and to avoid the increase of energy intake that often occurs when an untrained person starts to exercise [69]. On the other hand, we do not wish the participants to lose excessively mass due to reduced energy intake. To assess dietary habits during the course of the intervention, the participants fill out a food diary in the beginning, middle, and end of the intervention. Another possible limitation of the study may be a variation in the exercise training adherence. The major role of the personal trainers is to provide encouragement and support to the study participants. In addition, the researchers monitor the training adherence via PolarFlow service. However, it is expected that there are differences in the training adherence, and this will be taken into account when analyzing the results. The third limitation are the missing data points that are expected to occur due to problems in PET tracer production, malfunction of the imaging equipment, and other unforeseen events. To alleviate this major problem, missing data points are taken into account by restricted maximum likelihood estimation within the linear mixed models.

## Trial status

Ongoing. The first participants were enrolled in January 2019. The estimated date of the completion of the data collection is December 2021.

## Data Availability

The dataset generated and analysed during the current study are not publicly available in order to protect the individual privacy but are available, once the whole dataset is collected, from the corresponding author on reasonable request for researchers who have institutional review board/ethics approval and an institutionally approved study plan.

## Abbreviations

BMI: Body mass index
BSREM: Block sequential regularized expectation maximization
CT: Computed tomography
ECG: Electrocardiography
EDVI: End-diastolic volume index
ESVI: End-systolic volume index
FDG: 2-deoxy-2-[^18^F]fluoro-D-glucose
fMRI: Functional magnetic resonance imaging
GWAS: Genome wide association study
HbA1c: Glycated hemoglobin
HIIT: High intensity interval training
IAPS: International Affective Picture system
LA: Left atrium
LV: Left ventricle/ventricular
LVEF: Left-ventricular ejection fraction
MaRs-IB: Matrix reasoning item bank
MRI: Magnetic resonance imaging
MRS: Magnetic resonance spectroscopy
M-value: Whole-body insulin-stimulated glucose uptake
MZ: Monozygotic (twins)
OGTT: Oral glucose tolerance test
PCR: polymerase chain reaction
PET: positron emission tomography
PK11195: [1-(2-chlorophenyl)-N-methyl-N-(1-methylpropyl)-3-isoquinolinecarboxamide]
QCT: Quantitative computed tomography
rRNA: Ribosomal RNA
SD: Standard deviation
T2D: Type 2 diabetes
TDI: Tissue Doppler imaging
TSE: Turbo spin echo
TSOP: 18-kDA translocator protein
VO_2max_: Maximal oxygen uptake
WHO: World Health Organization
1RM: (External load that can be lifted once i.e. one repetition maximum)
2D: Two-dimensional
